# Comorbidity and polypharmacy impact neurobehavioral symptoms and symptom validity failure among post-9/11 veterans with mild traumatic brain injury

**DOI:** 10.3389/fneur.2023.1228377

**Published:** 2023-07-19

**Authors:** Alicia A. Swan, Eamonn Kennedy, Douglas B. Cooper, Megan E. Amuan, Jamie Mayo, David F. Tate, Kangwon Song, Blessen C. Eapen, Anne C. Van Cott, Maria R. Lopez, Mary Jo Pugh

**Affiliations:** ^1^Polytrauma Rehabilitation Center, South Texas Veterans Health Care System, San Antonio, TX, United States; ^2^Department of Psychology, The University of Texas at San Antonio, San Antonio, TX, United States; ^3^Veterans Affairs Salt Lake City Health Care System, Informatics Decision Enhancement and Analytic Sciences Center of Innovation, Salt Lake City, UT, United States; ^4^Division of Epidemiology, University of Utah School of Medicine Department of Internal Medicine, Salt Lake City, UT, United States; ^5^University of Utah School of Medicine Department of Neurology, Salt Lake City, UT, United States; ^6^Departments of Rehabilitation Medicine and Psychiatry, The University of Texas Health San Antonio, San Antonio, TX, United States; ^7^Augusta University Medical Center, Augusta University, Augusta, GA, United States; ^8^Department of Physical Medicine and Rehabilitation, VA Greater Los Angeles Healthcare System, Los Angeles, CA, United States; ^9^Division of Physical Medicine and Rehabilitation, Department of Medicine, David Geffen School of Medicine, University of California, Los Angeles, Los Angeles, CA, United States; ^10^Department of Neurology, University of Pittsburgh, Pittsburgh, PA, United States; ^11^Department of Veterans Affairs, Pittsburgh Healthcare System, Pittsburgh, PA, United States; ^12^Bruce Carter Hospital, Miami Veterans Health Administration, Miami, FL, United States; ^13^Miller School of Medicine, University of Miami, Miami, FL, United States

**Keywords:** veterans, polypharmacy, multimorbidity, post-traumatic stress disorders, concussion, headache

## Abstract

**Objective:**

The study aimed to examine the association between post-concussive comorbidity burdens [post-traumatic stress disorder (PTSD), depression, and/or headache] and central nervous system (CNS) polypharmacy (five or more concurrent medications) with reported neurobehavioral symptoms and symptom validity screening among post-9/11 veterans with a history of mild traumatic brain injury (mTBI).

**Setting:**

Administrative medical record data from the Department of Veterans Affairs (VA) were used in the study.

**Participants:**

Post-9/11 veterans with mTBI and at least 2 years of VA care between 2001 and 2019 who had completed the comprehensive traumatic brain injury evaluation (CTBIE) were included in the study.

**Design:**

Retrospective cross-sectional design was used in the study.

**Main measures:**

Neurobehavioral Symptom Inventory (NSI), International Classification of Diseases, Ninth Revision, and Clinical Modification diagnosis codes were included in the study.

**Results:**

Of the 92,495 veterans with a history of TBI, 90% had diagnoses of at least one identified comorbidity (PTSD, depression, and/or headache) and 28% had evidence of CNS polypharmacy. Neurobehavioral symptom reporting and symptom validity failure was associated with comorbidity burden and polypharmacy after adjusting for sociodemographic characteristics. Veterans with concurrent diagnoses of PTSD, depression, and headache were more than six times more likely [Adjusted odds ratio = 6.55 (99% CI: 5.41, 7.92)]. to fail the embedded symptom validity measure (Validity-10) in the NSI.

**Conclusion:**

TBI-related multimorbidity and CNS polypharmacy had the strongest association with neurobehavioral symptom distress, even after accounting for injury and sociodemographic characteristics. Given the regular use of the NSI in clinical and research settings, these findings emphasize the need for comprehensive neuropsychological evaluation for individuals who screen positively for potential symptom overreporting, the importance of multidisciplinary rehabilitation to restore functioning following mTBI, and the conscientious utilization of symptom validity measures in research efforts.

## Introduction

Traumatic brain injury (TBI) is a ‘signature wound of war' among post-9/11 veterans, with more than 463,000 documented cases since 2000 (1). Although 80–85% of all cases are mild in severity (mTBI), TBI of any severity can result in long-term changes in physical, emotional, and cognitive functioning (1). As a result, exposure to TBI is possibly better described as the potential onset of a multifaceted disease process rather than a historical event (2). Multiple, concurrent, chronic physical, or mental health conditions (i.e., multimorbidity) and/or central nervous system (CNS) polypharmacy (i.e., 5 or more central nervous system medications) can have compounding adverse effects on wellbeing and functioning (3–5). The challenges veterans of the post-9/11 era face encompass not only recovery but also the stress of stigma and the logistics of care for ambiguous and common clinical complaints, cumulatively described as the “burden of adversity.” (6).

The timely identification and treatment of TBI have the potential to prevent or delay the accumulation of related comorbidities, symptoms, and burdens (including CNS polypharmacy's adverse effects). In response, the Department of Veterans Affairs (VA) instituted mandatory TBI screening among all post-9/11 veterans in 2007 (7). Those who screen positively for TBI are referred for the comprehensive TBI evaluation (CTBIE), an in-depth structured interview, and physical assessment administered by a clinical specialist. Embedded within the CTBIE, the Neurobehavioral Symptom Inventory (NSI) assesses the impact of neurobehavioral symptoms commonly associated with TBI exposure (8).

TBI, however, is often followed by common mental and physical health symptoms, making it difficult to isolate TBI-related sequelae and symptoms from other causes. Given this complicated clinical picture, patients with a history of mTBI may present with an extensive medical history that includes treatment with multiple (sometimes concurrent) medications acting on the CNS (i.e., CNS polypharmacy) which can further exacerbate symptomology with medication-induced side effects (4, 9, 10).

To assess potential symptom overreporting, the Validity-10, an embedded measure of symptom validity, was developed leveraging low frequency or unusual symptom complaints on the NSI (11). Current guidance recommends that individuals who score 23 or greater on the Validity-10 should be referred for more comprehensive neuropsychiatric evaluation, presumably to evaluate psychological issues to which the symptom reporting might be better attributed. Its initial derivation, however, was in a sample of relatively healthy young men on active duty, from which those with neurological disorders were excluded. Although the Validity-10 is a valuable tool that has been praised for its clinical utility, concerns have emerged regarding its sensitivity among more clinically complex patient subgroups, particularly those with psychiatric conditions (12–14).

In this study, we examined the association between common comorbidities and polypharmacy with neurobehavioral symptom reporting among post-9/11 veterans with deployment-related mTBI. We first hypothesized that increasingly complex comorbidity would be associated with significantly greater neurobehavioral symptom burden and Validity-10 failure. Accounting for complex comorbidity, we also hypothesized that CNS polypharmacy would similarly have a significant association with increased neurobehavioral burden and Validity-10 failure. These findings are an important step toward increasingly sensitive estimates for symptom overreporting to account for the unique and complex needs of veterans experiencing multimorbidity associated with TBI exposure.

## Methods

### Sample

Following institutional review board approval, we used national health system data from the Departments of Defense (DoD) and Veterans Health Administration (VHA). The inclusion criteria were post-9/11 deployed veterans who a) had at least 3 years in the DoD (FY1999-FY2019) and b) at least 2 years of VHA care between fiscal year (FY) 2001 (1 October 2001) and the end of FY 2019 (30 September 2019). We further restricted this sample to those who completed the CTBIE after its institution in 2007 through 2018. The CTBIE is an in-depth clinical interview that includes a physical examination, a medical and psychiatric history, combat exposures, and the NSI. Finally, we limited the sample to veterans who were classified as having mTBI using a previously developed algorithm described elsewhere (15). For those who met these inclusion criteria, we compiled and merged VHA inpatient and outpatient healthcare data and pharmacy records from FY 2001 to FY 2020.

### Neurobehavioral symptom inventory

The NSI is a self-reported measure that assesses disruption over the past 30 days attributable to commonly observed symptoms after TBI (8). Response options for each item range from 0 (rarely or never present, not a problem at all) to 4 (almost always present, very severe problem) with total scores as the result of summing of all 22 items. Scores range between 0 and 88, where higher values indicate greater disruption of activities due to neurobehavioral symptoms. The NSI can also describe a more specific disruption due to affective, cognitive, somatosensory, and vestibular symptoms based on a recent factor analysis (16). Affective, cognitive, somatosensory, and vestibular subscale scores represent average responses to each item within the scale. To assess potential symptom overreporting, the Validity-10 was developed leveraging a subset of items of the NSI that are low frequency or unusual symptom complaints for healthy young active duty men (11). Scores of 23 or greater on the Validity-10 subscale are considered possible symptom overreporting, and clinical practice dictates that such patients should be referred for additional neuropsychiatric evaluation. Validity-10 status is therefore considered either “pass” (i.e., scores of 22 or lower) or “fail” (i.e., scores of 23 or greater).

### Comorbidity burden

Depression, headache, and PTSD were used to assess the comorbidity burden based on their prominent association with TBI among military veterans (10). We used the International Classification of Diseases, Ninth and Tenth Revisions, and Clinical Modification (ICD-9-CM and ICD-10-CM) diagnosis codes to identify PTSD, depression, and headache (see Supplementary Table 1) within 1 year (before or after) of the CTBIE. We used an established and conservative approach of requiring at least two diagnosis codes assigned at least 7 days apart in VA outpatient care or a single inpatient diagnosis to identify qualifying diagnoses (17). We then created a composite score based on the number of qualifying diagnoses among each PTSD, depression, and/or headache. The resultant variable was zero (“none”) for those with no history of PTSD, depression, or headache but otherwise equal to the count of these three diagnoses.

### CNS polypharmacy

The VHA Pharmacy Benefits Management outpatient database was used to identify unique outpatient medications dispensed by the VA product name. Although there is no standardized definition for polypharmacy, the field has most frequently utilized a definition of five or more concurrent CNS medications for younger patients (18). We used an algorithm to identify veterans in receipt of five or more CNS-active medications (hereafter polypharmacy, “yes” or “no”) within a year (before or after) of CTBIE administration (see Supplementary Table 2) (19).

### Sociodemographic characteristics

We collected personal and military history information from VHA datasets. From the CTBIE, we included age, sex (men or women), marital status (married/partnered, divorced/separated/widowed, or single/never married), race/ethnicity (white, Black Hispanic, Black non-Hispanic, Asian American and Pacific Islander [AAPI], Native American, Hispanic, or other), and education (high school or equivalent, college graduate/post-graduate, some college, less than high school, or unknown). Age violated the assumption of normality and was subsequently categorized into 19–29, 30–39, 40–50, or 51 years and older. We also classified veterans according to military branch (army, air force, marine corps, or navy/coast guard), component (active duty, national guard, or reserve), and rank (enlisted or officer).

### Statistical analyses

Descriptive statistics characterized the post-9/11 veteran sample by comorbidity burden. General linear modeling was used to predict NSI total and subscale (i.e., affective, cognitive, somatosensory, and vestibular) scores using comorbidity burden and polypharmacy variables while controlling for sociodemographic characteristics (age, sex, race/ethnicity, marital status, education, military branch, rank, and component). Model fits were measured by the coefficient of determination (*R*^2^). Logistic regression was used to predict Validity-10 failure using comorbidity burden and polypharmacy status while controlling for sociodemographic characteristics (age, sex, race/ethnicity, marital status, education, military branch, rank, and component). We used a *p*-value of < 0.01 as our level of significance and reported 99% confidence intervals for each analysis. All analyses were conducted using SAS^®^ Version 9.3 (SAS Institute, Cary, NC).

## Results

As shown in Table 1, on average, the sample was 31.9 (SD 8.2) years old, men (94%), white (63%), married/partnered (52%), and had a high school education or equivalent (60%). Most veterans were from the Army (72%) and served as enlisted members (96%). More than 28% of the sample met the criteria for polypharmacy. Approximately 91% of the sample had diagnoses of either PTSD, depression, headache, or some combination thereof diagnosed in VA care. Overall, 76.12% had diagnoses of PTSD, 53.59% depression, and 58.36% headache.

**Table 1:** Characteristics of the post-9/11 veteran sample with deployment-related mild traumatic brain injury (mTBI) by comorbidity burden.

***N*** **(%)**	**Comorbidity burden** ^*^				**Total sample** ***N* = 92,495**
	**None 7,664 (8.29)**	**One 22,894 (24.75)**	**Two 34,746 (37.57)**	**Three 27,191 (29.40)**	
Polypharmacy	183 (2.39)	2,419 (10.57)	9,857 (28.37)	13,536 (49.78)	25,995 (28.10)
**Age**					
19–29	4,191 (54.68)	12,519 (54.68)	17,631 (50.74)	12,033 (44.25)	46,374 (50.14)
30–39	1,951 (25.46)	6,287 (27.46)	10,846 (31.22)	9,431 (34.68)	28,515 (30.83)
40–49	1,157 (15.10)	3,205 (14.00)	5,004 (14.40)	4,686 (17.23)	14,052 (15.19)
50+	365 (4.76)	883 (3.86)	1,265 (3.64)	1,041 (3.83)	3,554 (3.84)
Sex (F)	241 (3.14)	933 (4.08)	1,681 (4.84)	2,228 (8.19)	5,083 (5.50)
**Race/ethnicity**					
White	5,203 (67.89)	15,269 (66.69)	22,119 (63.66)	16,028 (58.95)	58,619 (63.38)
Asian American/Pacific Islander	562 (7.33)	1,851 (8.09)	3,465 (9.97)	3,196 (11.75)	9,074 (9.81)
Black Hispanic	34 (0.44)	90 (0.39)	113 (0.33)	135 (0.50)	372 (0.40)
Black non-Hispanic	951 (12.41)	2,838 (12.40)	4,748 (13.66)	4,323 (15.90)	12,860 (13.90)
Hispanic	767 (10.01)	2,325 (10.16)	3,514 (10.11)	2,860 (10.52)	9,466 (10.23)
Native American	130 (1.70)	460 (2.01)	715 (2.06)	608 (2.24)	1,913 (2.07)
Unknown	17 (0.22)	61 (0.27)	72 (0.21)	41 (0.15)	191 (0.21)
**Marital status**					
Married/partnered	3,564 (46.50)	11,440 (49.97)	17,796 (51.22)	15,186 (55.85)	47,986 (51.88)
Divorced/separated/widowed	1,446 (18.87)	4,691 (20.49)	8,129 (23.40)	6,799 (25.00)	21,065 (22.77)
Single, never married	2,638 (34.42)	6,713 (29.32)	8,761 (25.21)	5,149 (18.94)	23,261 (25.15)
Unknown	16 (0.21)	50 (0.22)	60 (0.17)	57 (0.21)	183 (0.20)
**Education**					
High school or equivalent	4,486 (58.53)	13,723 (59.94)	21,314 (61.34)	16,149 (59.39)	55,672 (60.19)
College grad/post graduate	563 (7.35)	1,396 (6.10)	1,884 (5.42)	1,591 (5.85)	5,434 (5.87)
Less than high school	108 (1.41)	291 (1.27)	491 (1.41)	383 (1.41)	1,273 (1.38)
Some college	2,405 (31.38)	7,052 (30.80)	10,285 (29.60)	8,424 (20.98)	28,166 (30.45)
Unknown	102 (1.33)	432 (1.89)	772 (2.22)	644 (2.37)	1,950 (2.11)
**Military branch**					
Army	5,170 (67.46)	15,671 (68.45)	25,191 (72.50)	20,657 (75.97)	66,689 (72.10)
Air force	251 (4.58)	935 (4.08)	1,352 (3.89)	1,042 (3.83)	3,680 (3.98)
Marine corps	1,534 (20.02)	4,706 (20.56)	6,311 (18.16)	4,127 (15.18)	16,678 (18.03)
Navy/coast guard	609 (7.95)	1,582 (6.91)	1,892 (5.45)	1,365 (5.02)	5,448 (5.89)
**Military component**					
Active	2,694 (35.15)	8,592 (37.53)	14,929 (42.97)	14,293 (52.57)	40,508 (43.79)
Guard	1,421 (18.54)	3,955 (17.28)	6,035 (17.37)	4,454 (16.38)	15,865 (17.15)
Reserve	3,549 (46.31)	10,347 (45.20)	13,782 (39.66)	8,444 (31.05)	36,122 (39.05)
Military rank (enlisted**)**	7,235 (94.40)	21,926 (95.77)	33,525 (96.49)	26,144 (96.15)	88,830 (96.04)

mild traumatic brain injury, mTBI; central nervous system, CNS.

* The comorbidity burden value is defined as “none” for those with no history of PTSD, depression, or headache, but otherwise equal to the count of these three diagnoses.

### Total NSI and subfactor scores

Table 2 describes the mean scores on the NSI subscales (i.e., affective, cognitive, somatosensory, and vestibular) by comorbidity burden. The reported neurobehavioral symptom burden was the highest on the affective subscale and the lowest on the vestibular subscale. Regardless of the NSI subscale, the reported neurobehavioral symptom burden increased as the comorbidity burden increased.

**Table 2:** Mean neurobehavioral symptom inventory (NSI) scores by comorbidity burden.

**M (SD)**	**Comorbidity burden** ^*^			
	**None**	**One**	**Two**	**Three**
**NSI measure**				
Affective subscale	1.51 (0.88)	2.00 (0.95)	2.49 (0.87)	2.80 (0.81)
Cognitive subscale	1.36 (0.93)	1.73 (1.01)	2.13 (1.00)	2.45 (0.95)
Somatosensory subscale	0.91 (0.64)	1.21 (0.71)	1.46 (0.75)	1.75 (0.77)
Vestibular Subscale	0.78 (0.72)	1.00 (0.79)	1.24 (0.83)	1.49 (0.86)
Total NSI	25.46 (14.18)	33.13 (15.50)	40.66 (15.51)	47.01 (15.43)

* Comorbidity burden value is defined as “none” for those with no history of PTSD, depression, or headache but otherwise equal to the count of these three diagnoses.

As shown in Table 3, comorbidity burden and polypharmacy were each significantly associated with increased neurobehavioral symptom reporting. Comorbidity burden demonstrated an increasing association on all examined aspects of neurobehavioral symptom burden as the number of comorbid conditions increased, which was also the single largest association among all covariates in the analysis. Even after accounting for the effects of comorbidity burden, polypharmacy had a significant association with neurobehavioral symptom reporting, which was the second strongest association in the fully adjusted analysis.

**Table 3:** Results of the generalized linear model on each of the total score and subfactors of the Neurobehavioral Symptom Inventory (NSI).

**B, SE**	**Affective**	**Cognitive**	**Somatosensory**	**Vestibular**	**Total NSI**
	**R**^2^ = **0.21**	**R**^2^ = **0.14**	**R**^2^ = **0.16**	**R**^2^ = **0.10**	**R**^2^ = **0.20**
**Comorbidity burden** ^**^					
None	Ref	Ref	Ref	Ref	Ref
One	0.45 (0.01)^*^	0.33 (0.01)^*^	0.27 (0.01)^*^	0.19 (0.01)^*^	6.92 (0.20)^*^
Two	0.86 (0.01)^*^	0.66 (0.01)^*^	0.46 (0.01)^*^	0.37 (0.01)^*^	12.97 (0.19)^*^
Three	1.07 (0.01)^*^	0.88 (0.01)^*^	0.67 (0.01)^*^	0.54 (0.01)^*^	14.44 (0.20)^*^
**Polypharmacy**					
No	Ref	Ref	Ref	Ref	Ref
Yes	0.37 (0.01)^*^	0.37 (0.01)^*^	0.25 (0.01)^*^	0.26 (0.01)^*^	6.66 (0.12)^*^
**Age**					
19–29	Ref	Ref	Ref	Ref	Ref
30–39	0.07 (0.01)^*^	0.07 (0.01)^*^	0.10 (0.01)^*^	0.08 (0.01)^*^	1.57 (0.12)^*^
40–49	0.01 (0.01)	0.04 (0.01)^*^	0.19 (0.01)^*^	0.13 (0.01)^*^	2.00 (0.16)^*^
50+	0.02 (0.02)	0.07 (0.02)^*^	0.23 (0.01)^*^	0.26 (0.01)^*^	3.00 (0.27)^*^
**Sex**					
Men	Ref	Ref	Ref	Ref	Ref
Women	0.03 (0.01)	0.00 (0.01)	0.15 (0.01)^*^	0.16 (0.01)^*^	1.29 (0.22)^*^
**Race/ethnicity**					
White	Ref	Ref	Ref	Ref	Ref
Asian American/Pacific Islander	0.03 (0.01)^*^	0.03 (0.01)^*^	0.03 (0.01)^*^	0.04 (0.01)^*^	0.68 (0.18)^*^
Black Hispanic	0.17 (0.04)^*^	0.12 (0.05)^*^	0.17 (0.04)^*^	0.18 (0.04)^*^	3.35 (0.78)^*^
Black non-Hispanic	0.15 (0.01)^*^	0.04 (0.01)^*^	0.17 (0.01)^*^	0.12 (0.01)^*^	2.73 (0.15)^*^
Hispanic	0.07 (0.01)^*^	0.03 (0.01)	0.09 (0.01)^*^	0.09 (0.01)^*^	1.58 (0.17)^*^
Native American	0.07 (0.02)^*^	0.07 (0.02)^*^	0.09 (0.02)^*^	0.08 (0.02)^*^	1.70 (0.35)^*^
Unknown	0.09 (0.06)	−0.01 (0.07)	0.04 (0.05)	0.01 (0.06)	0.63 (1.08)
**Marital status**					
Married/partnered	Ref	Ref	Ref	Ref	Ref
Divorced/separated/widowed	0.04 (0.01)^*^	0.01 (0.01)	−0.01 (0.01)	−0.01 (0.01)	0.27 (0.12)
Single, never married	−0.09 (0.01)^*^	−0.09 (0.01)^*^	−0.11 (0.01)^*^	−0.08 (0.01)^*^	−2.01 (0.13)^*^
Unknown	−0.18 (0.06)^*^	−0.24 (0.07)^*^	−0.19 (0.05)^*^	−0.25 (0.06)^*^	−4.40 (1.13)^*^
**Education**					
High school or equivalent	Ref	Ref	Ref	Ref	Ref
College grad/post-graduate	−0.05 (0.01)^*^	−0.05 (0.02)^*^	0.00 (0.01)	−0.01 (0.01)	−0.68 (0.24)^*^
Less than high school	0.03 (0.02)	0.06 (0.03)	0.04 (0.02)	0.06 (0.02)^*^	1.04 (0.42)
Some college	−0.02 (0.01)^*^	−0.01 (0.01)	0.00 (0.01)	0.00 (0.01)	−0.17 (0.11)
Unknown	0.20 (0.02)^*^	0.24 (0.02)^*^	0.21 (0.02)^*^	0.23 (0.02)^*^	4.52 (0.35)^*^
**Branch**					
Army	Ref	Ref	Ref	Ref	Ref
Air force	−0.04 (0.01)	−0.05 (0.02)^*^	0.00 (0.01)	0.03 (0.01)	−0.45 (0.26)
Marine corps	−0.03 (0.01)^*^	−0.03 (0.01)^*^	0.00 (0.01)	0.00 (0.01)	−0.19 (0.14)
Navy/coast guard	−0.06 (0.01)^*^	−0.08 (0.01)^*^	−0.03 (0.01)	0.01 (0.01)	−1.04 (0.22)^*^
**Component**					
Active	Ref	Ref	Ref	Ref	Ref
Guard	−0.03 (0.01)^*^	−0.02 (0.01)	−0.04 (0.01)^*^	−0.05 (0.01)^*^	−0.64 (0.15)^*^
Reserve	−0.03 (0.01)^*^	−0.02 (0.01)	−0.05 (0.01)^*^	−0.05 (0.01)^*^	−0.69 (0.12)^*^
**Rank**					
Officer	Ref	Ref	Ref	Ref	Ref
Enlisted	0.11 (0.02)^*^	0.07 (0.02)^*^	0.10 (0.01)^*^	0.06 (0.02)^*^	2.06 (0.28)^*^

* Statistically significant at the *p* < 0 .01 level.

reference, Ref.

** The comorbidity burden value is defined as “none” for those with no history of PTSD, depression, or headache but otherwise equal to the count of these three diagnoses.

Older age, women (relative to men), veterans of non-white ethnicity (relative to white), married/partnered (relative to all other marital statuses), and enlisted service members (relative to officers) were each consistently associated with increased neurobehavioral symptom burden reporting. The various models ranged in variance were explained (*R*^2^) by the series of models, ranging from 10% (NSI-vestibular subscale) to 21% (NSI-affective subscale).

### Validity-10 failure

The results of the logistic regression predicting Validity-10 failure are shown in Table 4. Increasing comorbidity burden was associated with increased odds for Validity-10 failure, wherein those with PTSD, depression, and headache were more than six times more likely [Adjusted odds ratio = 6.55 (99% CI: 5.41, 7.92)]. to do so relative to those with none of those comorbidities. Similarly, those who met the criteria for polypharmacy were twice as likely to exceed the Validity-10 cutoff score. Consistent with the other NSI measures, older age, veterans of non-white ethnicity (relative to white), female veterans, and enlisted veterans (relative to officer) also had increased odds for Validity-10 failure.

**Table 4:** Results of the logistic regression predicting Validity-10 failure.

**AOR (99% CI)**	**Validity-10 failure**
**Comorbidity burden** ^**^	
None	Ref
One	2.50 (2.06, 3.04)^*^
Two	4.30 (3.55, 5.19)^*^
Three	6.55 (5.41, 7.92)^*^
**Polypharmacy**	
No	Ref
Yes	2.06 (1.95, 2.18)^*^
**Age**	
19–29	Ref
30–39	1.26 (1.18, 1.34)
40–49	1.53 (1.42, 1.66)^*^
50+	1.94 (1.71, 2.20)^*^
**Sex**	
Men	Ref
Women	1.20 (1.08, 1.33)^*^
**Race/ethnicity**	
White	Ref
Asian American/Pacific Islander	1.20 (1.10, 1.31)^*^
Black Hispanic	1.64 (1.14, 2.37)^*^
Black non-Hispanic	1.58 (1.47, 1.69)^*^
Hispanic	1.45 (1.33, 1.57)^*^
Native American	1.39 (1.18, 1.65)^*^
Unknown	0.95 (0.50, 1.80)
**Marital status**	
Married/partnered	Ref
Divorced/separated/widowed	0.99 (0.93, 1.06)
Single, never	0.82 (0.77, 0.89)^*^
Unknown	0.54 (0.28, 1.04)
**Education**	
High school or equivalent	Ref
College grad/post-graduate	1.00 (0.89, 1.13)
Less than high school	1.13 (0.91, 1.39)
Some college	0.99 (0.94, 1.05)
Unknown	1.58 (1.35, 1.85)^*^
**Military branch**	
Army	Ref
Air force	1.08 (0.95, 1.23)
Marine corps	1.03 (0.95, 1.11)
Navy/coast guard	0.98 (0.87, 1.10)
**Military component**	
Active	Ref
Guard	1.02 (0.95, 1.10)
Reserve	0.99 (0.93, 1.05)
**Military rank**	
Officer	Ref
Enlisted	0.76 (0.66, 0.89)^*^

* Statistically significant at the *p* < 0.01 level.

** The comorbidity burden value is defined as “none” for those with no history of PTSD, depression, or headache but otherwise equal to the count of these three diagnoses.

## Discussion

Among post-911 veterans with a history of mTBI, burdensome comorbidity, and CNS polypharmacy accounted for substantial variation in neurobehavioral symptom reporting after controlling for sociodemographic characteristics. This is consistent with previous studies, in that, while the NSI is a reliable and valid measure of post-concussive symptom distress, its scores are influenced by co-occurring psychiatric disorders such as PTSD, depression, and generalized anxiety (12, 20, 21). These analyses extend this effect to headache, a predominant complaint associated with TBI exposure. Perhaps more importantly, the Validity-10 failure rate was similarly affected by comorbidity burden and polypharmacy, revealing a need to account for TBI-related comorbidity in the utilization of the NSI. Patients with burdensome comorbidity following mTBI are likely best served by clinical and research contexts with increasingly holistic evaluation and care, particularly among those for whom potential symptom overreporting is a concern.

A history of mTBI exposure and psychological complaints are deeply and inextricably entwined (22). A clinical history of mTBI is emerging as a significant factor in the treatment for PTSD, influencing the type of treatment administered and how effective it may be (23, 24). It appears that, even though there are several treatments that are generally effective in treating PTSD symptoms, patients with a history of mTBI often have more persistent symptoms (25, 26). Given that Brenner et al. have similarly evidenced higher-than-expected rates of probable TBI among veterans seeking mental health services at the VA, a clearer understanding of how TBI and mental health are intertwined is critical (6). Therefore, although the Validity-10 symptom validity scale was initially derived from a clinically uncomplicated service member sample aside from deployment-related TBI status, its implementation could be adapted for use among the more complex patients often seen in polytrauma care. In fact, these findings emphasize the need for enhanced interdisciplinary clinical teams to ameliorate neurobehavioral symptoms and reduce polypharmacy (27). It is possible that clinical care teams with an eye for long-term adaptations in stress and mental health management could better enable sustainable reductions in symptom reporting and disability.

Increasingly advanced and sensitive statistical tools have recently made possible the identification of latent subgroups among clinic populations treating patients with TBI-related issues. For example, the polytrauma clinical triad describes a post-9/11 veteran patient subgroup that experienced TBI and grappled with pain and PTSD in its wake (28). Ensuing investigations have revealed that the constellation of clinical history and comorbid conditions encapsulated by the polytrauma clinical triad renders a substantially greater risk for prolonged symptomology, substance abuse, homelessness, and suicide-related behavior (3, 29, 30). Combined physical and health challenges experienced following TBI exposure can be enduring, progressive, and frequently diagnostically ambiguous, underscoring the need for an interdisciplinary approach to rehabilitation following TBI, regardless of its chronicity.

CNS polypharmacy status was associated with neurobehavioral symptom reporting, even when controlling for the comorbidity burden and sociodemographic characteristics. CNS polypharmacy is an established concern among older individuals as the risk for multimorbidity or multiple concurrent chronic conditions becomes more common in older age (31, 32). Older veterans appear to be at a particular risk for adverse outcomes associated with polypharmacy, including morbidity, mortality, and suicide (5, 19, 32). Moreover, polypharmacy itself has been repeatedly associated with a greater risk for suicide-related behaviors (4, 19, 33). Similar to the existing literature on CNS polypharmacy among older adults, post-911 veterans were also susceptible to severe adverse reactions secondary to having multiple CNS-acting medications on hand to manage co-existing chronic conditions. Side effects of common CNS depressants (e.g., somnolence, fatigue, and poor concentration) and/or CNS stimulants (e.g., headache, insomnia, dizziness, and dizziness) potentially amplified under conditions of polypharmacy and can be indistinguishable from otherwise diagnostically ambiguous TBI sequelae.^4(p)^ In fact, most patients subjected to polypharmacy are largely unaware of its existence or dangers, revealing that patient education may be an opportune path forward in ameliorating or preventing adverse outcomes to which this patient subpopulation may be particularly vulnerable (34). Our assessment of medication data in this effort is limited, however, to prescriptions filled at the VA and may underestimate the extent to which polypharmacy impacts military veterans. Fortunately, support for alternatives to pharmaceutical treatments for several post-concussive conditions (e.g., insomnia and headache), such as neurostimulation in its various incarnations, continues to mount in the literature (35–39). Given that more than 90% of the sample in the present study had mTBI and at least one other condition (i.e., PTSD, depression, and/or headache), the need for clinical practice guidelines sensitive to polypharmacy and multimorbidity is of great importance (40, 41).

Complex comorbidity and CNS polypharmacy impart a significant burden on the patient, provider, and healthcare system on the whole (41, 42). Heightened symptom reporting may influence clinical care pathways and affect patient engagement, particularly for racial/ethnic minorities or those with a lower socioeconomic status (43). In this analysis, minority status was associated with significantly greater neurobehavioral symptom distress and Validity-10 failure, even after accounting for many other socio = demographic and clinical characteristics. Although this effect was the most dramatic among Black Veterans, all minority veterans demonstrated a similar trend. Taken together with recent reports that extant barriers in the TBI screening process make the access and utilization of care more burdensome, renewed scrutiny and continued evolution of these programs are vital to enhancing long-term outcomes among veterans with mTBI (6, 9, 44). This finding may also underscore the continued need for diversity among clinical care providers, efforts to reduce stigma in the receipt of care, the enhancement of patient education efforts, and the coordination of complex care through social work and programmatic supports (45).

Consistent with the “burden of adversity” hypothesis, these findings have direct implications for the interpretation of neurobehavioral symptom burden scores captured by the NSI, particularly for patients with burdensome comorbidity (6). Landmark efforts to establish normative distributions (46) and develop the Validity-10 (11) for the NSI were undertaken among relatively homogenous clinical populations from which patients with neurological conditions were excluded. As a result, patients with more numerous and/or severe psychological, physical, or neurological comorbidities are not well characterized or represented by these initial efforts. Consistent with this gap, numerous reports have concluded that amended or refined thresholds for the Validity-10 may be critical to its implementation in more clinically complex populations (12, 13, 47–50). Moreover, the use of Validity-10 failure to exclude patients in research efforts may harm the generalizability of the findings and applicability to those most impacted by TBI-related neurobehavioral sequelae. Given that the potential symptom overreporting is presumably more consequential and prevalent in forensic settings (as opposed to clinical or research contexts, of which the VA is a unique entity given its role in service-connected disability adjudication), considering that the setting in which the NSI is collected could be a principal factor in its interpretation (51). Given that a recent report noted a stark dearth of effort assessments among veterans in receipt of diagnoses of early-onset dementia, a reminder of the need for its comprehensive assessment appeared warranted (52). Cumulatively, this underscores a need for more dynamic and comprehensive ways of evaluating symptom overreporting among patients with complex comorbidity who are more likely to indicate greater disruption from neurobehavioral symptoms by virtue of that comorbidity status.

This study has some notable limitations. First, while the examined factors sought to comprehensively capture clinical and sociodemographic factors associated with neurobehavioral symptom burden, the list is not necessarily exhaustive. These nationwide data were also cross-sectional and, therefore, only examined associations rather than causal relationships on the neurobehavioral symptom burden. Our approach to approximating comorbidity burden selected among the most common TBI-related sequelae (i.e., PTSD, depression, and headache) as an exhaustive list was considered less feasible and interpretable with the statistical tools available. Another limitation to using clinical diagnoses from ICD codes instead of diagnostic instruments is that clinicians tend to use heuristics to minimize the number of diagnoses and/or to ensure access to certain clinics. Assessment of medications was limited to those prescribed in the VA and did not include medications from outside providers or over-the-counter medications that may similarly contribute to polypharmacy status. Moreover, medication-level data in this evaluation were not considered in this analysis but could be an opportune future direction. The generalizability of the findings may be limited to veterans actively engaged in VHA care as care outside the VHA in more representative samples was not captured. Moreover, we included only post-9/11 veterans who completed the CTBIE in this analysis; thus, generalizability is limited to veterans who were screened and evaluated.

## Conclusion

This study sought to examine neurobehavioral symptom reporting among post-9/11 veterans with a history of deployment-related mTBI. Comorbidity burden (i.e., mTBI, PTSD, depression, and/or headache) and concomitant CNS polypharmacy provided significant explanative power in reported neurobehavioral symptom distress and the evaluation of symptom validity (i.e., Validity-10), underscoring a potential benefit for adjusted thresholds for clinically complex patients to maintain its specificity for identifying potential symptom overreporting. The application of the Validity-10 in complex clinical populations outside of mTBI likely extends the use of the brief screening measure beyond its original scope. As such, it is strongly recommended that “failure” on a single symptom validity assessment prompt the examination of additional indicators of effort and/or symptom distress. Patients reporting substantial distress on the NSI would likely benefit from treatment through a coordinated, multidisciplinary lens for clinical care. Additionally, careful consideration and implementation of symptom validity metrics in research efforts could improve representation for those with burdensome neurobehavioral symptoms that may have otherwise been excluded from analyses by virtue of that status. Consistent with the onus for and promising early evidence from the VA-funded Intensive Evaluation and Treatment Programs (IETP) project (53), there is a need for increasingly nuanced clinical practice guidelines and utility for interdisciplinary clinical teams to serve subpopulations of patients at a greater risk for multimorbidity or polypharmacy. Future efforts could expand on this line of inquiry to identify particularly deleterious combinations of CNS-active medications, particularly burdensome drug effects, or interactions that reveal opportunities for enhanced patient education and facilitate clinical care pathways that connect patients with clinical specialists in consideration of the totality of their rehabilitative journey.

Cumulatively, this study broadly invokes renewed consideration for the “burden of adversity,” a hypothesis that highlights the far-reaching effects of physical, mental, and functional disability that TBI-related multimorbidity can impart and highlights psychosocial, stigmatizing, and logistical challenges endemic to the pursuit of rehabilitation following TBI.

## Data availability statement

The datasets presented in this article are not readily available because the dataset is owned by the Department of Veterans Affairs and is subject to Federal restrictions. Requests to access the datasets should be directed to alicia.swan@va.gov.

## Ethics statement

The studies involving human participants were reviewed and approved by Salt Lake City VA IRB. Written informed consent for participation was not required for this study in accordance with the national legislation and the institutional requirements.

## Author contributions

AS, MP, EK, and MA contributed to conception and design of the study. MA organized the database and performed the statistical analysis. AS wrote the first draft of the manuscript. AS, MP, EK, JM, DC, AV, and ML wrote sections of the manuscript. All authors contributed to manuscript revision, read, and approved the submitted version.
