# Supplementary material for: Comorbidity and polypharmacy impact neurobehavioral symptoms and symptom validity failure among post-9/11 veterans with mild traumatic brain injury
Source: Front Neurol. 2023 Jul 19;14:1228377. doi: 10.3389/fneur.2023.1228377 (PMC10395329; doi:10.3389/fneur.2023.1228377)
Supplement: Supplementary file 1 [file Table_1.DOCX]

**Supplementary Material**

**Comorbidity and Polypharmacy Impact Neurobehavioral Symptoms and Symptom Validity Failure among Post-9/11 Veterans with Mild Traumatic Brain Injury**

Alicia A. Swan PhD^1,2*^; Eamonn Kennedy PhD^3,4,5^; Douglas B. Cooper PhD;^1,6^ Megan E. Amuan MPH; ^3,4^ Jamie Mayo PhD, PMHNP ^3,4^; David F. Tate PhD; ^3,5^ Kangwon Song PharmD;^7^ Blessen C. Eapen MD;^8,9^ Anne C. Van Cott MD;^10,11^ Maria R. Lopez, MD;^12,13^ and Mary Jo Pugh PhD RN^3,4^

**Supplemental Table 1.** International Classification of Diseases, Ninth Revision, Clinical Modification (ICD-9-CM) diagnosis codes by condition.

| **Condition** | **ICD-9-CM Code(s)** |
| --- | --- |
| Post-traumatic stress disorder (PTSD) | 309.81 |
| Depression | 296.2x, 296.3x, 311 |
| Headache | 307.81, 339.xx, 346.xx, 784.0 |

Abbreviations: ICD-9-CM, International Classification of Diseases, Ninth Revision, Clinical Modification; PTSD, post-traumatic stress disorder

**Supplemental Table 2.** Drug classification codes

| **Medication Type** | **Medication Class** | **VA drug classification code** |
| --- | --- | --- |
| CNS | Opioid analgesics | CN101 |
| CNS | Non-opioid analgesic | CN103 |
| CNS | Sedative-hypnotics | CN300, CN301, CN302, CN309 |
| CNS | Anti-depressants | CN601, CN602, CN609 |
| CNS | Anti-migraine agents | CN105 |
| CNS | Anticonvulsants | CN400 |
| CNS | Antiparkinson agents | CN500 |
| CNS | Antivertigo agents | CN550 |
| CNS | Antipsychotics, lithium | CN701, CN709, CN750 |
| CNS | Stimulants | CN801, CN802, CN809 |
| CNS | CNS, other | CN809 |
| Muscle Relaxants | Antispasticity, skeletal muscle relaxants | MS200, CV701 (metoxalone only) |

| **CN101. OPIOID ANALGESICS** |
| --- |
| ACETAMINOPHEN/BUTALBITAL/CAFFEINE/CODEINE  ACETAMINOPHEN/CODEINE  ACETAMINOPHEN/HYDROCODONE  ACETAMINOPHEN/OXYCODONE  ACETAMINOPHEN/PENTAZOCINE  ALFENTANIL  ASPIRIN/BUTALBITAL/CAFFEINE/CODEINE  ASPIRIN/CAFFEINE/DIHYDROCODEINE  ASPIRIN/OXYCODONE  BUPRENORPHINE  BUPRENORPHINE/NALOXONE  BUTORPHANOL  CODEINE  FENTANYL  HYDROCODONE/IBUPROFEN  HYDROMORPHONE  LEVORPHANOL  MEPERIDINE  METHADONE  MORPHINE  NALBUPHINE  NALOXONE/PENTAZOCINE  OXYCODONE  OXYMORPHONE  PENTAZOCINE  REMIFENTANIL  SUFENTANIL  TAPENTADOL |
| **CN102. OPIOID ANTAGONIST ANALGESICS (CN102 x 2)** |
| NALOXONE  NALTREXONE |
| **CN103. NON-OPIOID ANALGESICS** |
| ACETAMINOPHEN/ASPIRIN/CAFFEINE  ACETAMINOPHEN/BUTALBITAL/CAFFEINE  ASPIRIN/BUTALBITAL/CAFFEINE  CLONIDINE  TRAMADOL  TRAMADOL/ACETAMINOPHEN  ZICONOTIDE |
| **CN105. ANTIMIGRAINE AGENTS** |
| ACETAMINOPHEN/DICHLORALPHENAZONE/ISOMETHEPTENE  ALMOTRIPTAN  CAFFEINE/ERGOTAMINE  DIHYDROERGOTAMINE  ELETRIPTAN  FROVATRIPTAN  NARATRIPTAN  RIZATRIPTAN  SUMATRIPTAN  ZOLMITRIPTAN |
| **CN300. SEDATIVES/HYPNOTICS** |
| RAMELTEON |
| **CN301. BARBITURIC ACID DERIVATIVE SEDATIVES/HYPNOTICS** |
| AMOBARBITAL  PENTOBARBITAL  PHENOBARBITAL  SECOBARBITAL |
| **CN302. BENZODIAZEPINE DERIVATIVE SEDATIVES/HYPNOTICS** |
| ALPRAZOLAM  CHLORDIAZEPOXIDE  CLORAZEPATE  DIAZEPAM  ESTAZOLAM  FLURAZEPAM  LORAZEPAM  MIDAZOLAM  OXAZEPAM  TEMAZEPAM  TRIAZOLAM |
| **CN309. SEDATIVES/HYPNOTICS,OTHER** |
| BUSPIRONE  CHLORAL HYDRATE  DEXMEDETOMIDINE  ESZOPICLONE  MEPROBAMATE  ZALEPLON  ZOLPIDEM |
| **CN400. ANTICONVULSANTS** |
| CARBAMAZEPINE  CLONAZEPAM  DIAZEPAM  DIVALPROEX  ETHOSUXIMIDE  FELBAMATE  FOSPHENYTOIN  GABAPENTIN  LACOSAMIDE  LAMOTRIGINE  LEVETIRACETAM  METHSUXIMIDE  OXCARBAZEPINE  PERAMPANEL  PHENYTOIN  PRIMIDONE  TIAGABINE  TOPIRAMATE  VALPROATE SODIUM  VALPROIC ACID  ZONISAMIDE |
| **CN500. ANTIPARKINSON AGENTS** |
| APOMORPHINE  CARBIDOPA/ENTACAPONE/LEVODOPA  CARBIDOPA/LEVODOPA  ENTACAPONE  PRAMIPEXOLE  RASAGILINE  ROPINIROLE  ROTIGOTINE  SELEGILINE  TOLCAPONE |
| **CN550. ANTIVERTIGO AGENTS** |
| MECLIZINE  SCOPOLAMINE |
| **CN601. TRICYCLIC ANTIDEPRESSANTS** |
| AMITRIPTYLINE  AMOXAPINE  CLOMIPRAMINE  DESIPRAMINE  DOXEPIN  IMIPRAMINE  NORTRIPTYLINE  PROTRIPTYLINE  TRIMIPRAMINE |
| **CN602. MONAMINE OXIDASE INHIBITOR ANTIDEPRESSANTS** |
| ISOCARBOXAZID  PHENELZINE SULFATE  SELEGILINE  TRANYLCYPROMINE |
| **CN609. ANTIDEPRESSANTS,OTHER** |
| BUPROPION  CITALOPRAM  DESVENLAFAXINE  DULOXETINE  ESCITALOPRAM  FLUOXETINE  FLUVOXAMINE  MAPROTILINE  MILNACIPRAN  MIRTAZAPINE  NEFAZODONE  PAROXETINE  SERTRALINE  TRAZODONE  VENLAFAXINE  VILAZODONE |
| **CN701. PHENOTHIAZINE/RELATED ANTIPSYCHOTICS** |
| CHLORPROMAZINE  FLUPHENAZINE  PERPHENAZINE  THIORIDAZINE  THIOTHIXENE  TRIFLUOPERAZINE |
| **CN709. ANTIPSYCHOTICS,OTHER** |
| ARIPIPRAZOLE  CLOZAPINE (CLOZARIL)  CLOZAPINE (FAZACLO)  CLOZAPINE (MYLAN)  HALOPERIDOL  LOXAPINE  LURASIDONE  OLANZAPINE  PALIPERIDONE  QUETIAPINE  RISPERIDONE  ZIPRASIDONE |
| **CN750. LITHIUM SALTS** |
| LITHIUM |
| **CN801. AMPHETAMINES** |
| AMPHETAMINE RESIN COMPLEX  AMPHETAMINE/DEXTROAMPHETAMINE  DEXTROAMPHETAMINE  LISDEXAMFETAMINE |
| **CN802. AMPHETAMINE LIKE STIMULANTS** |
| METHYLPHENIDATE |
| **CN809. CNS STIMULANTS,OTHER** |
| ARMODAFINIL  CAFFEINE/SODIUM BENZOATE  MODAFINIL |
| **CN900. CNS MEDICATIONS,OTHER** |
| ACETAMINOPHEN/DIPHENHYDRAMINE  ALCOHOL  AMITRIPTYLINE/CHLORDIAZEPOXIDE  AMITRIPTYLINE/PERPHENAZINE  ATOMOXETINE  DEXTROMETHORPHAN/QUINIDINE  DONEPEZIL  ERGOLOID MESYLATES  FLUOXETINE/OLANZAPINE  GALANTAMINE  MEMANTINE  PIMOZIDE  **PREGABALIN**  RILUZOLE  RIVASTIGMINE  SODIUM OXYBATE |
| **MS200. SKELETAL MUSCLE RELAXANTS AND ANTISPASTICITY AGENTS** |
| BACLOFEN  DANTROLENE  CARISOPRODOL  CHLORZOXAZONE  CYCLOBENZAPRINE  METOXALONE  METHOCARBAMOL  ORPHENADRINE  TIZANADINE |

**Note:** New medications may be added since the development of this list, but are included in the drug class.
